# The effect of capsule tension ring on posterior capsule opacification: A meta-analysis

**DOI:** 10.1371/journal.pone.0246316

**Published:** 2021-03-15

**Authors:** Kaikai Zhang, Yuchen Dong, Meisheng Zhao, Lili Nie, Xinfen Ding, Chao Zhu

**Affiliations:** Ophthalmology, The Second Affiliated Hospital of Jilin University, Changchun, China; Faculty of Medicine, Cairo University, EGYPT

## Abstract

**Background:**

Posterior capsule opacification is one of the most common complications after cataract surgery. Studies have suggested that the introduction of a capsule tension ring might play a critical role in the prevention of capsule opacification, yet quantitative evidence is still lacking. This work consists of a meta-analysis on available data in order to explore the influence of a capsule tension ring on posterior capsule opacification.

**Methods:**

A comprehensive review of the literature on capsule tension ring and posterior capsule opacification was carried out using the Embase, Pubmed, Web of Science, and Cochrane electronic databases. The selected studies included randomized controlled trials, retrospective studies and prospective studies published before June 2020. The studies of interest were selected by two reviewers independently from the included studies. Odds ratios (ORs) and standardized mean differences (SMD) were used in order to assess the association. A fixed-effects model or a random-effects model was applied to combine data according to heterogeneities. Sensitivity analysis was used to assess the heterogeneity of the studies. Publication bias was estimated using the Egger test. Statistical analysis was performed using the stata15.1 software.

**Results:**

The meta-analysis included in total 8 studies involving 379 cases and 333 controls. There was a statistically significant difference of Nd:YAG laser capsulotomy rate (OR=0.241, 95% CI: 0.145, 0.400 I^2^=42.1%) between the capsule tension ring group and the control group, indicating that the tension ring reduced the Nd:YAG laser capsulotomy rate. Further studies with continuous data also revealed that the use of capsule tension ring was associated with a lower posterior capsule opacification score (SMD = -1.402, 95% CI: -2.448, -0.355 I^2^=95.0%). The sensitivity analysis suggested that the result of the re-combined analysis did not change notably, indicating that the result was reliable and stable. Both pooled analysis showed no evidence of publication bias.

**Conclusion:**

The findings of this meta-analysis confirmed that capsule tension ring might reduce capsule opacification. Further studies should be made to validate the result.

## Introduction

Posterior capsule opacification(PCO) is the most common postoperative complication after cataract surgery and can often cause vision loss [1]. The visual acuity loss due to posterior capsule opacification accounts for 20-40 percent of all patients, 2 to 5 years after cataract surgery and has become the second most common cause of visual loss [1,2]. Posterior capsule opacification development is attributed to the residual lens epithelial cells (LECs) in the capsular bag after cataract surgery that experience proliferation, migration, metaplasia, differentiation and opacification [3–5]. Nd:YAG capsulotomy is currently the only effective treatment of PCO [6]. However, the measure could potentially cause vision-threatening complications including increasing intraocular pressure, IOL damage, cystoid macular edema and retinal detachment [7]. Currently, a number of techniques have been used clinically to prevent posterior capsule opacification that include improving surgical techniques, different IOL materials and designs or administrating specific pharmacological agents [8–12].

Leger and Witschel in 1993, were the first to implant a capsule tension ring into the human eye during a cataract surgery. The ring was inserted to maintain postoperatively the shape of the capsular bag in case of zonular weakness or loss [13]. During a complex cataract surgery, zonular weakness is treated with the help of a capsule tension ring that improves safety and success [14,15]. In the case of zonular dehiscence or defect, however, cataract surgeons are often unable to comply adequate hydrodissection and effective cortical aspiration leading to lens epithelial cells remaining longer in the capsular bag. Residual lens epithelial cells lead to an increased chance of posterior capsule opacification. The use of a capsule tension ring is reported to not only stabilize the capsular bag and IOL position, but also to significantly reduce posterior capsule opacification [16,17].

Despite the fact that the benefit of a capsule tension ring in the reduction of posterior capsule opacification has been reported in a several studies [18–20]. There still has not been a meta-analysis on the association between the use of a capsule tension ring and posterior capsule opacification. Therefore, this study aims to quantify the effect capsule tension ring on posterior capsule opacification.

## Materials and methods

### Search strategy and study selection

The meta-analysis was done strictly based on the Cochrane Handbook for Systematic Reviews and Meta-Analysis (PRISMA) guidelines [21]. The PubMed, Web of Science, Embase, and Cochrane databases were searched systematically for related studies that were published before June 2020 for the search terms “equator rings”, “tension rings”, “bending rings”, “capsule opacification”. In the first instance, selection of the studies was done on the basis of titles and abstracts by two reviewers (KKZ and YCD) and, subsequently, the reviewers screened the full text using the inclusion criteria. The references of selected papers were hand-searched further to expand the study sample. Any issues regarding study selection were resolved with the assistance of the third author (MSZ).

### Inclusion criteria

The selected studies had to meet the following inclusion criteria: (1) studies on the association between the use of a capsule tension ring a**n**d posterior capsule opacification; (2) study designs were limited to randomized controlled trial, retrospective study and prospective analysis; (3) intervention: IOL and CTR were implanted in capsular bag during cataract surgery; control: only IOL was implanted in capsular bag during cataract surgery; (4) original studies quantitatively reporting the posterior capsule opacification outcome.

### Exclusion criteria

(1) unavailability of the selected studies;(2) studies without controlled group;(3) intraocular lens was implanted into capsule bag. (4) studies were in vitro. (5) meeting, abstract, review, case report.

### Data extraction and quality assessment

Two reviewers (KKZ and YCD) independently collected the relevant data based on a standardized data extraction form. The collected content includes the first author’s name, publication date, country, sample size, study design, major outcome (PCO score and Nd:YAG laser capsulotomy rate). The Newcastle-Ottawa scale (NOS) was used to determine the non-randomized study’s quality [22]. The stars that met at least five were deemed to be high quality. Any issues were settled through consultation with the third author (MSZ).

### Statistical analysis

Stata15.1 software was used to perform all the statistical analysis. Categorical data, such as the Nd: YAG laser capsulotomy rate, were pooled in odds ratio (OR) with corresponding 95% confidence intervals (CI). Continuous data, such as the PCO score, were analyzed using mean differences (MD) with 95% CI. The I^2^ value was used to describe heterogeneity among studies. When heterogeneity was observed (I^2^>50%), the random-effect model was used; otherwise, the fixed-effect model was used. Moreover, a sensitivity analysis was carried out to evaluate the stability of the meta-analysis estimates. The Egger test assessed the potential publication bias [23]. Significance level was set at P < 0.05.

## Results

**Fig 1:**
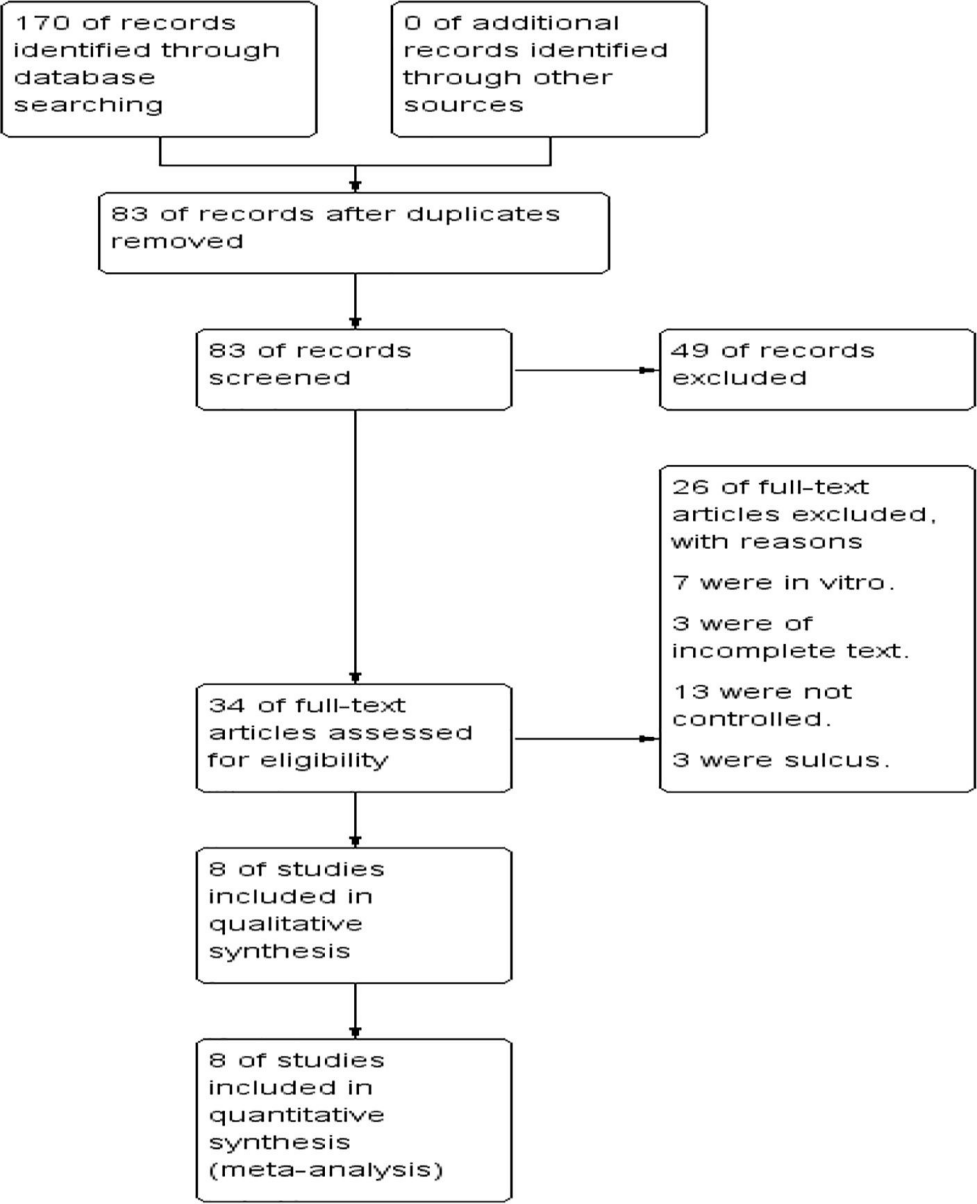
Flow diagram.

The complete procedure of study selection is presented in Fig 1. A total of 170 articles were identified using the four academic databases, of which 87 records were eliminated as duplicates. Subsequently, 49 citations were removed after evaluating their titles and abstracts. The residual 34 full-text essays were further evaluated for their eligibility. Among them, 26 articles were excluded for the following reasons: 7 studies were in vitro, 3 were of incomplete text, 13 were not controlled and 3 were inserted in sulcus. Finally, 8 items [24–31] met the inclusion criteria and were included for quantitative analysis. Selected studies involved in total 379 cases and 333 controls. The sample size ranged from 44 to 153. There were 8 articles published between 2001 and 2014. Among the induced studies, 5 were from Asia, and 3 were from Europe. Table 1 summarizes the characteristics of the selected studies and the score of the quality assessment.

**Table 1:** Characteristics of the selected studies and quality assessment score.

Author	Year	Country	Sample size		Outcome data	Design	Score
			Cases	controls			
Keles	2014	Turkey	78	75	Capsulotomy rate	Retro	8
Halili	2012	Albania	34	34	Capsulotomy rate, PCO score	Pro	8
Kim	2005	Korea	41	38	Capsulotomy rate	Retro	7
Menapace	2008	Austria	50	50	Capsulotomy rate, PCO score	Pro	9
Nishi	2001	Japan	42	38	Capsulotomy rate, PCO score	Pro	8
Takimoto	2008	Japan	47	34	Capsulotomy rate, PCO score	Retro	8
Eliseo	2002	Italy	65	62	Capsulotomy rate	Retro	6
Zhao	2009	China	22	22	Capsulotomy rate, PCO score	Pro	6

Pro: Prospective study Retro: Retrospective study.

### Statistical analysis

**Fig 2:**
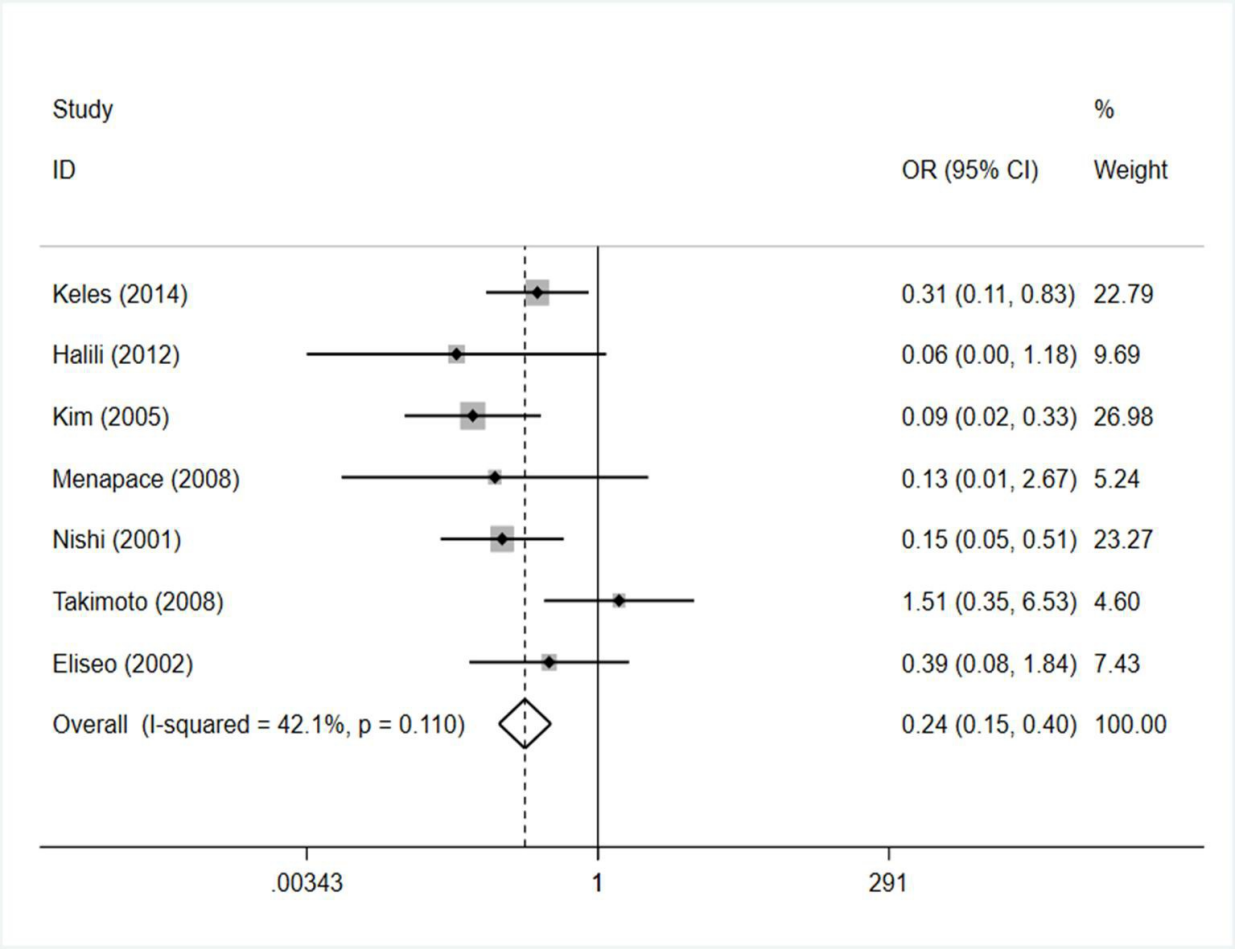
Forest plot of the effect of capsule tension ring on capsulotomy rate.

Nd:YAG laser capsulotomy rate: The meta-analysis included a total of 7 studies involving 357 cases and 311 controls [24–31]. No significant heterogeneity was discovered in the combined analysis (I^2^ = 42.1%), therefore a fixed-effect model was chosen to reduce heterogeneity. According to the pooled results, the capsule tension ring patients exhibited a lower Nd:YAG laser capsulotomy rate than the control group. (OR=0.241, 95% CI: 0.145, 0.400 I^2^=42.1%) (Fig 2).

**Fig 3:**
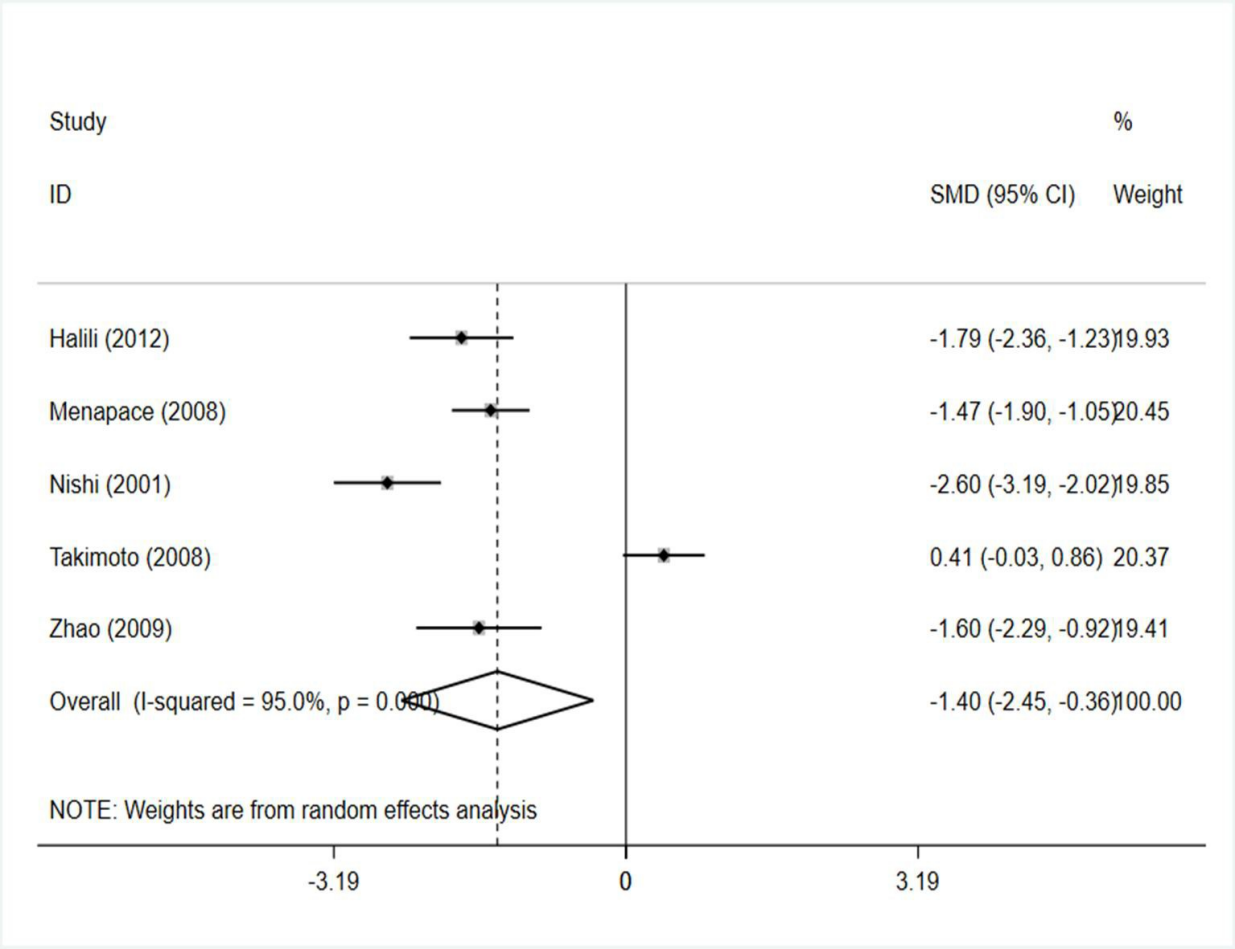
Forest plot of the effect of a capsule tension ring on PCO score.

PCO score: The meta-analysis contained a total of 5 studies involving 195 cases and 192 controls [25,27–29,31]. Obvious heterogeneity was detected in the pooled analysis (I^2^ = 95.0%), so a randomized effect model was applied to reduce errors. The pooled results revealed that the capsule tension ring patients had lower PCO scores than the control group (SMD = -1.402, 95% CI: -2.448, -0.355 I^2^=95.0%) (Fig 3).

### Sensitivity analysis and publication bias

**Fig 4:**
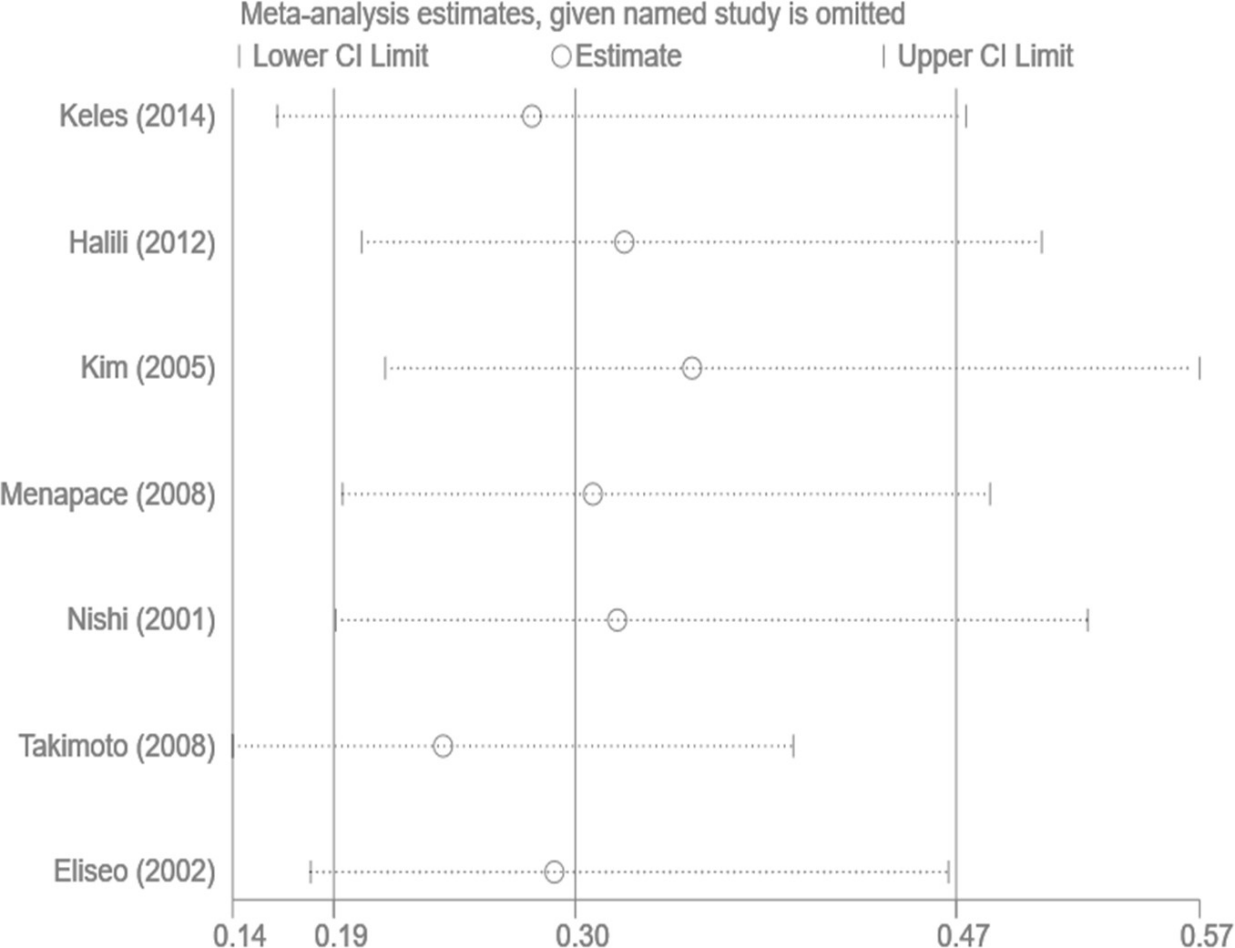
Sensitivity analysis for capsule on capsulotomy rate.

**Fig 5:**
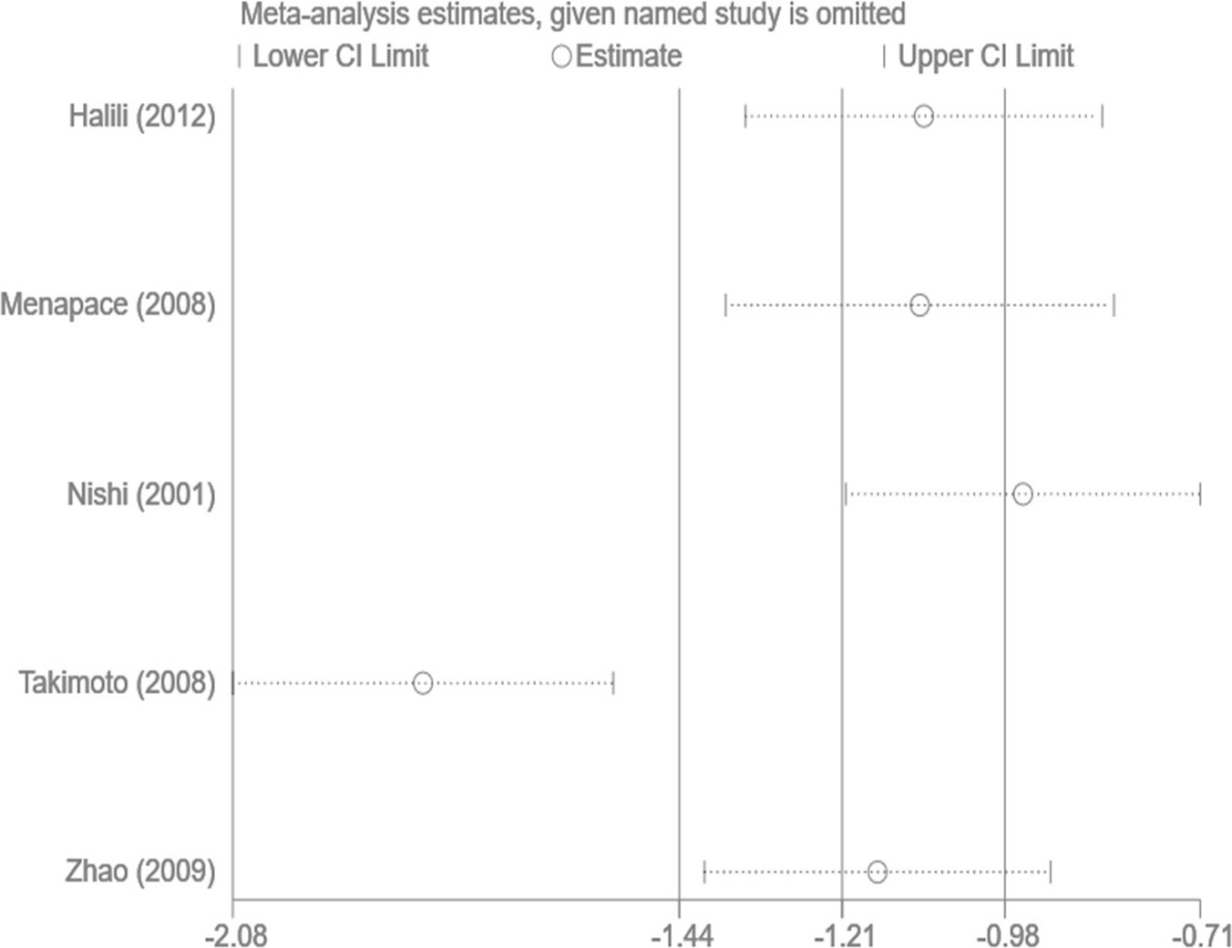
Sensitivity analysis for capsule on PCO score.

Sensitivity analysis was performed by sequentially omitting single studies. The heterogeneity was similar before and after the study exclusion, indicating that the meta-analysis was stable and reliable. The Egger test showed no clear publication bias in the analysis of the Nd:YAG laser capsulotomy rate (Egger test *P* =0.981) and the PCO score (Egger test *P* =0.355) (Figs 4 and 5).

## Discussion

Cataract surgery with zonular deficiency increased the risk of capsular tears, IOL decentration, vitreous loss [32]. Formerly, cataract with zonular weakness considered extracapsular cataract extraction, intracapsular cataract extraction or pars plana lensectomy. So, Hara firstly described capsule tension ring to support zonular comprise in 1991 [33]. A train of studies had investigated that capsule tension ring not only increased the safety of cataract surgery, but also reduced posterior capsule opacification [34–37]. Hence, we gathered the related studies and executed the meta-analysis to define the value of capsule tension ring on PCO.

Our meta-analysis consisted of 8 studies involving 379 cases and 333 controls, revealing that capsule tension ring was beneficial to reduce capsulotomy rate and PCO score. When PCO score was combined, there was significant heterogeneity in pooled results and, therefore, a randomized-effect model was chosen to reduce heterogeneity. Sensitivity analysis showed that the result of the re-combined analysis did not change significantly, indicating that the result was stable. The Egger test did not find visible publication bias in the analysis of Nd:YAG laser capsulotomy rate and the PCO score. In the past, cataract surgery with zonular weakness often did not clear cortex which caused the rising of posterior capsule opacification. Some studies found that capsule tension ring not only enhanced the safety of cataract surgery, but also reduced posterior capsule opacification. In addition, studies indicated that capsule tension ring also increased the stability of intraocular lens and vision [38].

There are several candidates to explain the mechanisms of capsule opacification reduction when using a capsule tension ring: first, it is thought to reduce largely the lens-capsule distance and make cortical clean-up easier during cataract surgery by posterior capsule stretching [39–41]. Second, the uniform and discontinuous capsule bending along the optical edge is believed to inhibit the migration of LECs [42,43]. Finally, the ring is thought to move the anterior capsular leaf away from the anterior optic surface and the posterior capsule [44].

It needs to be noted, however, that in this work there are several points to be considered: first, the content of the research was restricted solely to published studies, and therefore articles not yet published or gray literature with a possibility of satisfying inclusion criteria were not included. Also, the pooled results exhibited heterogeneity; the sensitivity analysis still did not reveal the source of this heterogeneity and subgroup analysis and meta-regression were not conducted further. Finally, the included samples only contained 712 patients which meant potential publication bias.

## Conclusions

In summary, it was found out that the use of a capsule tension ring in cataract surgery leads to a less Nd:YAG laser capsulotomy rate and PCO score. However, further research using larger samples, multiple centers, and high-quality studies are necessary to verify our results.

## Supporting information

S1 Checklist PRISMA checklist. (DOC)

S1 File Quality assessment. (DOCX)
