# Supplementary material for: The effect of capsule tension ring on posterior capsule opacification: A meta-analysis
Source: PLoS One. 2021 Mar 15;16(3):e0246316. doi: 10.1371/journal.pone.0246316 (PMC7959402; doi:10.1371/journal.pone.0246316)
Supplement: S1 File — (DOCX) [file pone.0246316.s002.docx]

| Study | Selection |  |  |  | Comparability |  | Outcome |  |  | Score |
| --- | --- | --- | --- | --- | --- | --- | --- | --- | --- | --- |
| Keles | 1 | 1 | 1 | 1 | 1 | 1 | 1 | 1 | 0 | 8 |
| Halili | 1 | 1 | 1 | 1 | 1 | 1 | 1 | 1 | 0 | 8 |
| Kim | 1 | 1 | 1 | 1 | 1 | 0 | 1 | 1 | 0 | 7 |
| Menapace | 1 | 1 | 1 | 1 | 1 | 1 | 1 | 1 | 1 | 9 |
| Nishi | 1 | 1 | 1 | 1 | 1 | 0 | 1 | 1 | 1 | 8 |
| Takimoto | 1 | 1 | 1 | 1 | 1 | 1 | 1 | 1 | 0 | 8 |
| Eliseo | 1 | 1 | 1 | 1 | 0 | 0 | 1 | 1 | 0 | 6 |
| Zhao | 1 | 1 | 1 | 1 | 0 | 0 | 1 | 1 | 0 | 6 |
